# Alternative formulations and improved bounds for the multi-depot fleet size and mix vehicle routing problem

**DOI:** 10.1007/s00291-017-0494-y

**Published:** 2017-11-12

**Authors:** Rahma Lahyani, Leandro C. Coelho, Jacques Renaud

**Affiliations:** 1 Alfaisal University, College of Business, Riyadh, Kingdom of Saudi Arabia; 2 Interuniversity Research Center on Enterprise Network, Logistics and Transportation, Quebec City, QC Canada; 3 Faculté des sciences de l’administration, Université Laval, Quebec City, Canada; 4 Canada Research Chair in Integrated Logistics, Quebec City, Canada; 5 Faculty of Economics and Business, Groningen University, Groningen, the Netherlands; 6 LOGIQ Laboratory, Institut Supérieur de Gestion Industrielle, Sfax, Tunisia

**Keywords:** Vehicle routing problem, Multi-depot, Heterogeneous fleet, Formulation, Mathematical model, Exact solution

## Abstract

In this paper, we compare different formulations of the multi-depot fleet size and mix vehicle routing problem (MDFSMVRP). This problem extends the multi-depot vehicle routing problem and the fleet size and mix vehicle routing problem, two logistics problems that have been extensively studied for many decades. This difficult vehicle routing problem combines complex assignment and routing decisions under the objective of minimizing fixed vehicle costs and variable routing costs. We first propose five distinct formulations to model the MDFSMVRP. We introduce a three-index formulation with an explicit vehicle index and a two-index formulation in which only vehicle types are identified. Other formulations are obtained by defining aggregated and disaggregated loading variables. The last formulation makes use of capacity-indexed variables. For each formulation, we summarize known and propose new valid inequalities, including symmetry breaking, lexicographic ordering, routing, and rounded capacity cuts. We then implement branch-and-cut and branch-and-bound algorithms for these formulations, and we fed them into a general purpose solver. We compare the bounds provided by the formulations on a commonly used set of instances in the MDFSMVRP literature, containing up to nine depots and 360 customers, and on newly generated instances. Our in-depth analysis of the five formulations shows which formulations tend to perform better on each type of instance. Moreover, our results have considerably improved available lower bounds on all instances and significantly improved quality of upper bounds that can be obtained by means of currently available methods.

## Introduction

Distribution problems are central to the performance of many industries. The area of transportation has been widely studied, notably the vehicle routing problem (VRP) (Toth and Vigo 2014) which has attracted the interest of many researchers for more than 50 years (Laporte 2009) and is still among the most prominent and widely studied combinatorial optimization problems. Several different exact and heuristic algorithms have been proposed since the seminal paper of Dantzig et al. (1954), and in the past decade a myriad of practical applications have emerged, describing many variants of the classical capacitated VRP (Lahyani et al. 2015b; Coelho et al. 2016). These variants often incorporate ad hoc decisions or constraints to address challenging problems observed from practice.

In this paper, we model and solve the multi-depot fleet size and mix vehicle routing problem (MDFSMVRP). This problem is a direct generalization of the classical VRP by considering multiple depots and different types of vehicles to serve a set of customers with known demands. It combines three decisions simultaneously: selecting the number of vehicles of each type, planning vehicle routes and assigning routes to depots. Each vehicle is characterized by a fixed and a variable cost proportional to the traveled distance. The number of available vehicles of each type is assumed to be unlimited. The simultaneous optimization of the best fleet composition, the best vehicle routes and the depot choice substantiates the challenges of this problem. The MDFSMVRP consists of designing a set of vehicle routes, each starting and ending at the same depot, visiting each customer exactly once, and respecting the capacity of the vehicles. The objective is to minimize the total fixed and variable routing costs.

The literature dealing explicitly with the MDFSMVRP is not well furnished as the problem includes several prominent problems as special cases, such as the multi-depot VRP (MDVRP) and the FSMVRP. We are aware of four works focusing on the MDFSMVRP. A seminal work on this specific variant is due to Salhi and Sari (1997). The authors propose a multi-level composite heuristic based on integrating and modifying efficient heuristics designed for the single depot fleet size and mix vehicle routing problem (FSMVRP). Their method relies on switching to a more powerful and expensive neighborhood when moving to a superior level. The authors integrate reduction tests and refinement modules in the heuristic to speed up some of its steps. Seventeen years later, Salhi et al. (2014) propose a mixed integer linear program to formulate the problem and a set of valid inequalities to tighten it. They also propose a variable neighborhood search metaheuristic. The method distinguishes between customers served from their nearest depots and borderline customers. It makes use of local search heuristics and Dijkstra’s algorithm to refine the tours constructed for each depot separately. The authors derive lower and upper bounds using a 3-h execution of CPLEX and provide percentage gaps computed using the best known bounds. Recently, Vidal et al. (2014) propose a unified algorithmic framework tackling different classes of multi-depot VRPs with and without fleet mix. They present two metaheuristics, i.e., a multi-start iterated local search and a hybrid genetic algorithm. Both methods rely on a bidirectional dynamic programming approach to efficiently evaluate customer-to-depots assignments. The three published works described above assess the performance of their methods on the same testbed. Finally, Mancini (2016) tackles a real and new variant of the MDFSMVRP with multiple periods and different levels of incompatibility between customers and vehicles, and between customers and days. The author proposes an adaptive large neighborhood search metaheuristic and demonstrates the performance of her method on a new set of randomly generated instances.

Many papers and book chapters have been devoted to study separately the two straightforward reductions of the MDFSMVRP. The primary decision related to the fleet consists to determine the optimal size of the fleet, which may be limited for the heterogeneous fleet vehicle routing problem (HFVRP) or unlimited for the FSMVRP. Dealing with the fleet composition goes back to the seminal papers by Gheysens et al. (1984) and Golden et al. (1984). Since then, several variants of the FSMVRP have been addressed in the literature, including those with time windows (Koç et al. 2015), pickups and deliveries (Qu and Bard 2014), multi-trip (Prins 2009), and more recently green routing (Juan et al. 2014; Saka et al. 2017), and multi-compartment (Derigs et al. 2011; Lahyani et al. 2015a). Several metaheuristics (Euchi and Chabchoub 2010; Liu 2013) addressed large-scale instances of the FSMVRP, while exact methods (Yaman 2006; Baldacci and Mingozzi 2009) manage smaller-size ones. For more details, we refer to recent surveys proposed by Baldacci et al. (2008), Andersson et al. (2010), Irnich et al. (2014a) and Koç et al. (2016b).

The literature for the MDVRP is also rich. This problem first appeared in the literature in the works of Kulkami and Bhave (1985) and Carpaneto et al. (1989). More intricate and extended variants of the MDVRP have also been studied. Mancini (2016) and Rahimi-Vahed et al. (2015) consider a closely related problem with multiple periods. Time-related constraints including static and fuzzy time windows for customers and depots have also received increased attention in the last few years, e.g., Bettinelli et al. (2011), Xu and Jiang (2014), Adelzadeh et al. (2014) and Koç et al. (2016a) along with the consideration of open vehicles routes, e.g., Liu et al. (2014), Lalla-Ruiz et al. (2015). Besides, several works focused on studying the MDVRP combined with the location problem, e.g., Contardo et al. (2013) and Prodhon and Prins (2014). The great amount of solution methods proposed for the MDVRP has been focused on the design of metaheuristic algorithms, e.g., genetic algorithms (Thangiah and Salhi 2001; Vidal et al. 2012) and adaptive large neighborhood search algorithm (Pisinger and Ropke 2007) for large-scale cases as well as exact algorithms (Baldacci and Mingozzi 2009; Contardo et al. 2013; Contardo and Martinelli 2014) for smaller scale instances. An extensive survey of all methods is outside the scope of this section, and we refer to Karakatič and Podgorelec (2015) and Montoya-Torres et al. (2015) for focused and recent surveys for the MDVRP. In Karakatič and Podgorelec (2015) the authors focused on reviewing genetic algorithms designed for solving MDVRPs and compared them to other existing approaches, both exact and heuristic for solving this same problem. In Montoya-Torres et al. (2015) the authors presented a systematic review of MDVRP literature and an analysis of both single- and multiple-objective multi-depot problems.

The MDFSMVRP is an NP-hard combinatorial problem since the VRP is NP-hard. Several authors explicitly outline the difficulty of solving to optimality either the FSMVRP instances or the MDVRP instances, or even finding tight bounds (Pessoa et al. 2009; Salhi et al. 2014).

Our contributions lie in adapting and proposing new formulations and valid inequalities for the MDFSMVRP. Specifically, we propose a model based on a three-index VRP formulation introduced by Laporte and Nobert (1987), to which we include new dimensions to account for each vehicle type, as in Vidal et al. (2014). We then present a formulation derived from the two-index VRP model of Laporte (1986), in which we create copies of the graph for each vehicle type, but we do not identify individual vehicles. Our third formulation is derived from the commodity flow model proposed in Salhi and Rand (1993) and Yaman (2006), which we modify to consider multiple depots. This formulation makes use of loading variables to model capacity and subtour elimination constraints. We obtain our fourth formulation by disaggregating the loading variables by vehicle type, as in Yaman (2006). Finally, the last formulation we propose is derived from the model of Pessoa et al. (2009) for the FSMVRP, which is compact enough to enumerate all variables and constraints, and to which we incorporate new procedures to reduce the number of variables. We compare these five formulations in order to provide tighter bounds for this difficult routing problem. The focus of the paper is on the last three compact formulations since the first two path-based formulations have been extensively studied in the VRP literature. We compare the last three formulations against the one proposed by Salhi et al. (2014). The five formulations are intended to be solved with a general purpose solver. We also describe the thinking path and the conceptual motivation behind the proposition of the five formulations. A subproduct of this research is to identify the origins and give credits to the main ideas used by our community to formulate many distribution problems. Thus, for each proposed formulation, we provide the main references that have put forward the modeling techniques and the valid inequalities used.

The remainder of this paper is organized as follows. In Sect. 2, we provide a formal description of the MDFSMVRP, followed by the introduction of the five mathematical models. Section 3 presents a description and discussion of several theoretical analysis for the VRP and lays down some theoretical comparison for our five models. The algorithms used to solve these formulations are briefly presented in Sect. 4. The results of extensive computational experiments are presented in Sect. 5. Section 6 is devoted to our conclusions.

## Problem description and mathematical formulations

The MDFSMVRP is formally defined on a directed graph ${\mathcal {G}} = ({\mathcal {V}}, {\mathcal {A}})$, where ${\mathcal {V}}$ is the vertex set and ${\mathcal {A}}$ is the arc set. The vertex set ${\mathcal {V}}$ is partitioned into two subsets ${\mathcal {V}}_d = \{1, \ldots , m\}$ representing *m* depots, and ${\mathcal {V}}_c =\{m+1, \ldots , m+n\}$ representing *n* customers, such that ${\mathcal {V}} = {\mathcal {V}}_d \cup {\mathcal {V}}_c$. Each customer $i \in {\mathcal {V}}_c$ is associated with a nonnegative demand $q_i$, while $q_i = 0$, $i \in {\mathcal {V}}_d$. The distance between nodes *i* and $j \in {\mathcal {V}}$ is $\beta _{{ij}}$. In the following, we use the terms cost and distance interchangeably. The use of loop arcs (*i*, *i*) is not allowed including loop arcs between customers $i \in {\mathcal {V}}_c$ and loop arcs between depots $i \in {\mathcal {V}}_d$. This is imposed by defining $\beta _{ii}=\infty$ for all $i \in {\mathcal {V}}$. Also, back-and-forth arcs connecting two different depots are forbidden by imposing $\beta _{{ij}}=\infty$ for all $i,j \in {\mathcal {V}}_d, i\ne j$. Graph ${\mathcal {G}}$ is assumed to be complete as it includes all the arcs connecting the vertex pairs, with the exception of loops. If the distance matrix is asymmetric, the arc set ${\mathcal {A}}$ is composed of $\{(i,j) : i, j \in {\mathcal {V}}, i\ne j\}$. Otherwise, i.e., when $\beta _{{ij}}=\beta _{{ji}}$, the arc set ${\mathcal {A}}$ is replaced by a set of undirected edges, ${\mathcal {E}}$ such that ${\mathcal {E}}$ is composed of edges $\{(i,j) : i, j \in {\mathcal {V}}, i>j \}$. A fleet of heterogeneous vehicles ${\mathcal {K}} = \{1, \ldots , K\}$ with different capacities and housed in multiple depots $d \in {\mathcal {V}}_d$ is available. Each vehicle type $k \in {\mathcal {K}}$ is associated with a capacity $Q^k$, a fixed cost $F^k$ and a variable cost $\alpha ^k$ per unit of distance. Each vehicle must start and end its journey at the same depot. We define a set ${\mathcal {H}}$ as a fleet including *n* copies of each vehicle type *k*, with $|{\mathcal {H}}|=nK$. Each vehicle has an index $t \in {\mathcal {H}}$ that represents its number. Each vehicle $t \in {\mathcal {H}}$ is associated with a capacity $Q^t$, a fixed cost $F^t$ and a variable cost $\alpha ^t$ which are equal to the capacity $Q^k$, the fixed cost $F^k$, and the variable cost $\alpha ^k$ of its corresponding type *k*.

**Fig. 1:**
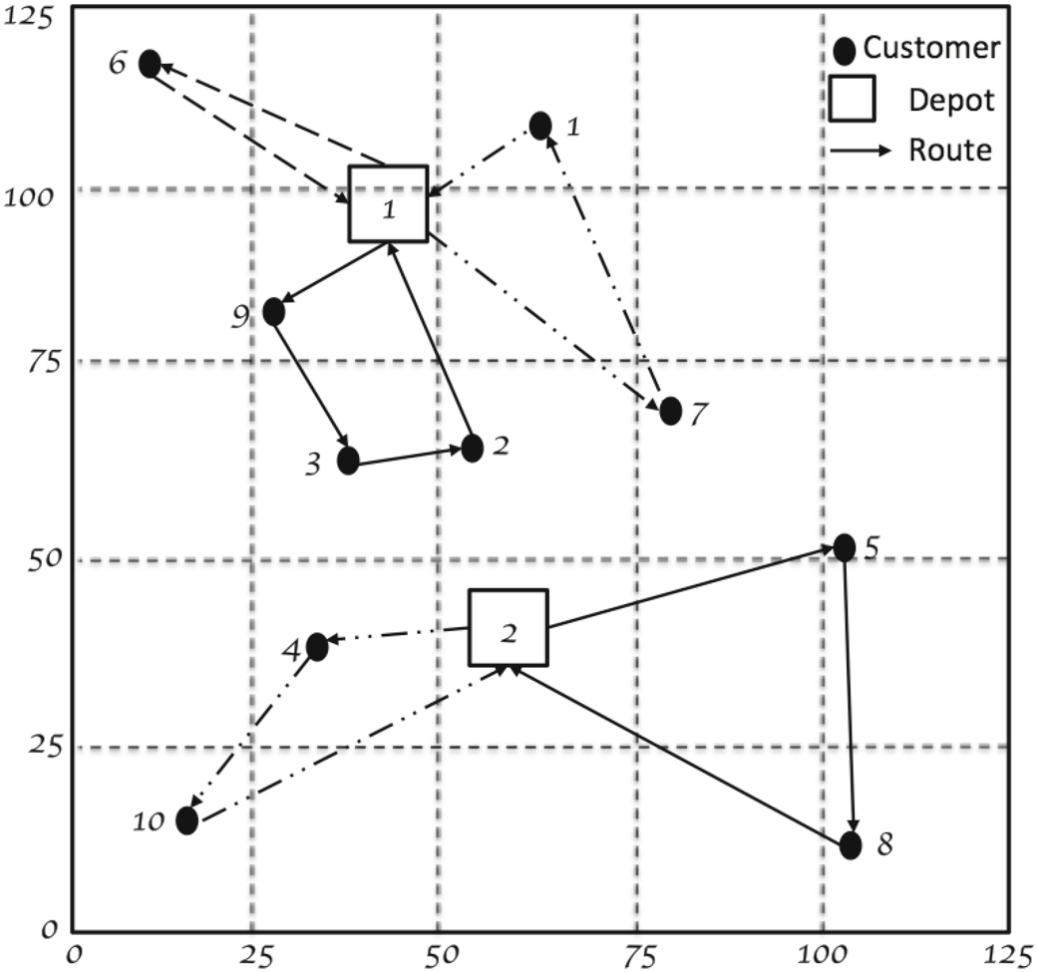
Solution for the MDFSMVRP with 2 depots, 10 customers and 3 vehicle types

A solution to the problem must determine routes that minimize the total costs such that each route must start and end at the same depot, each customer is visited exactly once, and the total demand of each route does not exceed the capacity of the selected vehicle. Also, when solving this problem, the vehicle fleet composition will be found for all depots as well as for each depot. An illustration of this problem is shown in Fig. 1 where each route is performed by a different vehicle.

We now provide five different formulations for the MDFSMVRP. In Sect. 2.1 we present a vehicle flow model which explicitly considers all edges, vehicles and depots. In Sect. 2.2 we show an adaptation of the two-index formulation, notably extending it to handle an heterogeneous fleet. Section 2.3 presents a commodity flow formulation in which capacity and subtour elimination constraints are expressed using flows. In Sect. 2.4 we introduce a model based on disaggregated loading variables by vehicle type. In Sect. 2.5 we present a capacity-indexed formulation for the problem at hand.

### Explicit formulation

We first provide a three-index vehicle flow formulation for the symmetric case with an explicit vehicle index. The extension to an asymmetric version is straightforward. This formulation is based on the model proposed by Golden et al. (1977) for the multiple depot vehicle routing problem with length restrictions, on the three-index vehicle flow formulation proposed by Laporte and Nobert (1987) for the asymmetrical multi-depot VRP with homogeneous fleet, on the model proposed by Toth and Vigo (2002) for the single depot VRP, and on the model proposed by Vidal et al. (2014) for the MDFSMVRP. Note that as required for this symmetric case, the arc set ${\mathcal {A}}$ is replaced by the set of undirected edges, ${\mathcal {E}}=\{(i,j) : i, j \in {\mathcal {V}}, i>j \}$, because as it was stated in Toth and Vigo (2002) and Irnich et al. (2014b), it is not necessary to know in which direction edges are traversed by the vehicles. We define routing variables $x_{{ij}}^{{td}}$ to indicate the number of times (0, 1, 2) edge $(i,j) (i>j )$ is used in the solution. $x_{{ij}}^{{td}}$ equal to one if edge (*i*, *j*) is traversed by vehicle *t* housed at depot *d*, and equal to two for a round trip to customer *j*. Binary variables $y_i^{{td}}$ are equal to one if node *i* is visited by vehicle *t* from depot *d*. Note that, as in Vidal et al. (2014), in formulation F1 *t* refers to the vehicle index, not the vehicle type since all the available vehicles are explicitly considered. However, unlike Vidal et al. (2014), we define different assignment variables $y_i^{{td}}$. The problem can then be formulated as follows:

1 $$\begin{aligned} {\text {(F1) minimize}} \sum _{i\in {\mathcal {V}}_d} \sum _{t\in {\mathcal {H}}} \sum _{d\in {\mathcal {V}}_d} F^t {y_{i}^{{td}}} + \sum _{(i,j)\in {\mathcal {E}}} \sum _{t\in {\mathcal {H}}} \sum _{d\in {\mathcal {V}}_d} {\alpha ^t \beta _{{ij}}}{x_{{ij}}^{{td}}} \end{aligned}$$

subject to

2 $$\begin{aligned} \sum _{t\in {\mathcal {H}}} \sum _{d\in {\mathcal {V}}_d} {y_{i}^{{td}}}= & {} 1 \quad i\in {\mathcal {V}}_c \end{aligned}$$

3 $$\begin{aligned} \sum _{j\in {\mathcal {V}}, i>j} x_{i j}^{{td}} + \sum _{j\in {\mathcal {V}}, j>i} x_{j i}^{{td}}= & {} 2 y_{i}^{{td}} \quad i\in {\mathcal {V}}, t\in {\mathcal {H}}, d\in {\mathcal {V}}_d \end{aligned}$$

4 $$\begin{aligned} y_{i}^{{td}}\le & {} y_{d}^{{td}} \quad i\in {\mathcal {V}}_c, t\in {\mathcal {H}}, d\in {\mathcal {V}}_d \end{aligned}$$

5 $$\begin{aligned} y_{d}^{{td}}\le & {} \sum _{(i,j)\in {\mathcal {E}}} x_{i j}^{{td}} \quad t\in {\mathcal {H}}, d\in {\mathcal {V}}_d \end{aligned}$$

6 $$\begin{aligned} 2y_{d}^{{td}}\le & {} \sum _{i\in {\mathcal {V}}_c} x_{id}^{{td}} \quad t\in {\mathcal {H}}, d\in {\mathcal {V}}_d \end{aligned}$$

7 $$\begin{aligned} \sum _{i\in \mathcal {Z}} \sum _{j\in \mathcal {Z}, i>j} x_{{ij}}^{{td}}\le & {} \sum _{i\in \mathcal {Z}} y_{i}^{{td}} - y_{z}^{{td}} \quad \mathcal {Z} \subseteq {\mathcal {V}}_c, \vert \mathcal {Z}\vert \ge 2, z\in \mathcal {Z}, t\in {\mathcal {H}}, d\in {\mathcal {V}}_d\nonumber \\ \end{aligned}$$

8 $$\begin{aligned} \sum _{i\in {\mathcal {V}}_c} q_{i} y_{i}^{{td}}\le & {} Q^{t} \quad t\in {\mathcal {H}}, d\in {\mathcal {V}}_d \end{aligned}$$

9 $$\begin{aligned}&x_{i j}^{{td}} \in \{0,1\} \quad (i,j)\in {\mathcal {E}}, t\in {\mathcal {H}}, d\in {\mathcal {V}}_d \end{aligned}$$

10 $$\begin{aligned}&x_{i j}^{{td}} \in \{0,1,2\} \quad i\in {\mathcal {V}}_c, j\in {\mathcal {V}}_d, t\in {\mathcal {H}}, d\in {\mathcal {V}}_d \end{aligned}$$

11 $$\begin{aligned}&y_{i}^{{td}} \in \{0,1\} \quad i\in {\mathcal {V}}, t\in {\mathcal {H}}, d\in {\mathcal {V}}_d. \end{aligned}$$

The objective function (1) minimizes the total cost composed of fixed vehicle costs and variable routing costs. Constraints (2) impose that all customers must be visited exactly once. Constraints (3) are degree constraints and constraints (4) impose that if a customer is served by vehicle *t* housed at depot *d*, then vehicle *t* must leave the depot. Constraints (5) and (6) link the two types of variables of the problem. They ensure that if a vehicle *t* of depot *d* is used, then at least one customer *i* must be visited by this vehicle. Constraints (7) forbid subtours. Constraints (8) impose vehicle capacities. The domain of the variables is enforced by constraints (9)–(11). This formulation contains $O(2^n)$ subtour elimination constraints whose number grows exponentially with *n*. This is a large formulation which strongly depends on the number of available vehicles.

Model F1 is sufficient to represent the MDFSMVRP; however, we can add some valid inequalities and lift some constraints to strengthen it. Constraints (12) enforce restrictions related to the vehicle use. Specifically, each vehicle *t* housed at depot *d* is allowed to perform at most one trip.

12 $$\begin{aligned} \sum _{j\in {\mathcal {V}}_c} x_{j d}^{{td}} \le 2 \quad t\in {\mathcal {H}}, d\in {\mathcal {V}}_d. \end{aligned}$$

To avoid symmetries due to the presence of identical vehicles at each depot, we introduce vehicle symmetry breaking constraints. Observe that (13) and (14) are only valid if the fleet is homogeneous. We define the set ${\mathcal {H}}^k\subset {\mathcal {H}}$ containing only the homogeneous vehicles of type *k*. Thus, constraints (13) state that vehicle *t* can only be dispatched if vehicle $t-1$ is already dispatched. Constraints (14) rank identical vehicles according to the index of the customers visited. These constraints are defined for each depot. They are inspired by those presented in Adulyasak et al. (2013), Coelho and Laporte (2013), Coelho and Laporte (2014), and Lahyani et al. (2015a).

13 $$\begin{aligned} y_{d}^{{td}}\le & {} y_{d}^{t-1,d} \quad t\in {\mathcal {H}}^k \backslash \{{\mathcal {H}}^k_1\}, {\mathcal {H}}^k \subset {\mathcal {H}}, k \in {\mathcal {K}}, d\in {\mathcal {V}}_d \end{aligned}$$

14 $$\begin{aligned} y_{i}^{{td}}\le & {} \sum _{j\in {\mathcal {V}}_c, j<i} \sum _{h\in {\mathcal {V}}_d} y_{j}^{t-1,h} \quad i\in {\mathcal {V}}_c, t\in {\mathcal {H}}^k \backslash \{{\mathcal {H}}^k_1\}, {\mathcal {H}}^k \subset {\mathcal {H}}, k \in {\mathcal {K}}, d\in {\mathcal {V}}_d,\nonumber \\ \end{aligned}$$

where ${\mathcal {H}}^k_1$ represents the first element of ${\mathcal {H}}^k.$

We also introduce a set of logical inequalities that enforce the relationships between routing and visiting variables. They are defined as follows:

15 $$\begin{aligned}&y_{d}^{{td}} \le \sum _{i\in {\mathcal {V}}_c} y_{i}^{{td}} \quad t\in {\mathcal {H}}, d\in {\mathcal {V}}_d \end{aligned}$$

16 $$\begin{aligned}&x_{id}^{{td}} \le 2 y_{i}^{{td}} \quad i\in {\mathcal {V}}_c, t\in {\mathcal {H}}, d\in {\mathcal {V}}_d \end{aligned}$$

17 $$\begin{aligned}&x_{{ij}}^{{td}} \le y_{j}^{{td}} \quad i, j\in {\mathcal {V}}_c, i>j, t\in {\mathcal {H}}, d\in {\mathcal {V}}_d \end{aligned}$$

18 $$\begin{aligned}&\sum _{j\in {\mathcal {V}}_c} y_{j}^{{td}} \le \sum _{(i,j)\in {\mathcal {E}}} x_{{ij}}^{{td}} \quad t\in {\mathcal {H}}, d\in {\mathcal {V}}_d \end{aligned}$$

19 $$\begin{aligned}&2y_{j}^{{td}} \le \sum _{i\in {\mathcal {V}}_c} x_{id}^{{td}} \quad j\in {\mathcal {V}}_c, t\in {\mathcal {H}}, d\in {\mathcal {V}}_d \end{aligned}$$

20 $$\begin{aligned}&\sum _{j\in {\mathcal {V}}, i>j} \sum _{t\in {\mathcal {H}}}\sum _{d\in {\mathcal {V}}_d} x_{i j}^{{td}} + \sum _{j\in {\mathcal {V}}, i<j} \sum _{t\in {\mathcal {H}}}\sum _{d\in {\mathcal {V}}_d} x_{j i}^{{td}} = 2 \quad i\in {\mathcal {V}}_c \end{aligned}$$

21 $$\begin{aligned}&\left\lceil \frac{\sum _{i\in {\mathcal {V}}_c} q_i }{\max \{Q^t\}}\right\rceil \le \sum _{i\in {\mathcal {V}}_c} \sum _{t\in {\mathcal {H}}} \sum _{d\in {\mathcal {V}}_d} x_{id}^{{td}}. \end{aligned}$$

Constraints (15)–(20) are referred to as routing cuts. The first ones replace the right hand side of constraints (5) by enforcing that at least one customer must be visited by vehicle *t* of depot *d* if this vehicle is used. We also note that constraints (15) are the sum over the customers in inequalities (4). Constraints (16) remove all edges (*i*, *d*) if customer *i* is not visited by vehicle *t* of depot *d*. Constraints (17) further remove variables by forbidding the use of edge (*i*, *j*) if customer *j* is not visited by vehicle *t* housed at depot *d*. Constraints (18) impose that the sum of customers visited by vehicle *t* is less than or equal to the sum of edges traversed by vehicle *t*. Constraints (19) impose the condition that if vehicle *t* of depot *d* is not used, then customer *j* cannot be visited by this vehicle. Equations (20) further define the degree constraints by imposing that each customer is visited once. Finally, constraints (21) are referred to as rounded capacity cuts (Naddef and Rinaldi 2002; Pessoa et al. 2009). They impose a lower bound on the number of used vehicles. However, in the case it is not necessary to use the vehicle with the biggest capacity in one trip, and if there is a considerable difference between $\max \{Q^t\}$ and the capacity of the used vehicle, then the left hand side of constraints (21) may give a poor lower bound. Even if these constraints are redundant in this context, they are known to help the mathematical programming solvers derive new cuts and improve the overall algorithmic performance (Coelho and Laporte 2014; Gendron and Crainic 1994; Jena et al. 2015b, a; Lahyani et al. 2015a).

Constraints (22) and (23) are lexicographic ordering constraints. They are inspired from the ones defined in Sherali and Smith (2001) and Adulyasak et al. (2013). Given the large coefficients that arise when dealing with large instances, these constraints are only added for small and medium size instances containing up to 60 customers.

22 $$\begin{aligned} \sum _{i=m+1}^{j} 2^{(j-i)}y_{i}^{{td}}\le & {} \!\!\!\sum _{i=m+1}^{j} 2^{(j-i)}y_{i}^{t-1,d} \, j\in {\mathcal {V}}_c, t\in {\mathcal {H}}^k \backslash \{{\mathcal {H}}^k_1\}, {\mathcal {H}}^k \subset {\mathcal {H}}, k \in {\mathcal {K}}, d\in {\mathcal {V}}_d\nonumber \\ \end{aligned}$$

23 $$\begin{aligned} \sum _{i\in {\mathcal {V}}_c} 2^{(m+n-i)} y_{i}^{{td}}\le & {} \sum _{i\in {\mathcal {V}}_c} 2^{(m+n-i)}y_{i}^{t-1,d} \quad t\in {\mathcal {H}}^k \backslash \{{\mathcal {H}}^k_1\}, {\mathcal {H}}^k \subset {\mathcal {H}}, k \in {\mathcal {K}}, d\in {\mathcal {V}}_d.\nonumber \\ \end{aligned}$$

### Implicit vehicle index formulation

Formulation F1 has the drawback that the number of variables and constraints increases when the number of vehicles increases. These variables are dependent on the number of customers *n*.

We now propose a formulation with implicit vehicle assignment as proposed in Laporte (1986), and Toth and Vigo (2002) for the single depot VRP.

This formulation uses the same type of variables defined in Sect. 2.1 but using the index *k* instead of *t* which refers to vehicle types instead of individual vehicles. This has the advantage of having one type of variable per vehicle type, instead of creating one variable per vehicle of each type. For the sake of briefness, we do not restate the whole definition of the variables and constraints (in this section and for the remainder of this paper), and refer to the ones already defined in Sect. 2.1 when the interpretation is straightforward. The only difference consists in to replace parameter *t* by parameter *k* while defining the variables and the sets. This implicit vehicle index formulation can then be defined as follows:

24 $$\begin{aligned} {\text {(F2) minimize}} \sum _{j\in {\mathcal {V}}_c} \sum _{k\in {\mathcal {K}}} \sum _{d\in {\mathcal {V}}_d} 0.5 F^k {x_{jd}^{{kd}}} + \sum _{(i,j)\in {\mathcal {E}}} \sum _{k\in {\mathcal {K}}} \sum _{d\in {\mathcal {V}}_d} {\alpha ^k \beta _{{ij}}}{x_{i j}^{{kd}}} \end{aligned}$$

subject to (2)–(6), (9)–(11) and to

25 $$\begin{aligned} \sum _{i\in {\mathcal {S}}} \sum _{j\in {\mathcal {S}}, i>j} {x_{i j}^{{kd}}} \le |{\mathcal {S}}|- r({\mathcal {S}}) \quad {\mathcal {S}} \subseteq {\mathcal {V}}_c, {\mathcal {S}} \ne \emptyset , k\in {\mathcal {K}}, d\in {\mathcal {V}}_d. \end{aligned}$$

When using a compact variables definition, the objective function (24) is expressed by the variables $x_{i j}^{{kd}}$. Constraints (25) simultaneously replace constraints (7) and (8) where $r({\mathcal {S}})$ is a lower bound on the number of vehicles required to serve the customers in ${\mathcal {S}}$. They correspond to generalized subtour elimination constraints, and prevent capacity violation on each vehicle. This formulation has a number of linear subtour elimination constraints growing exponentially with *n*. F2 is much more compact than F1 since it depends on the number of vehicle types.

Several of the valid inequalities previously defined remain valid, namely (15)–(21). Alternatively, we can reinforce subtour elimination (25) by introducing inequalities (7). Constraints (7) are known to be efficient when solving the problem with a branch-and-cut algorithm. Both families of constraints (7) and (25) have a cardinality growing exponentially with *n*.

Vehicle symmetry breaking constraints and lexicographic ordering constraints no longer hold for this formulation because they require distinguishing between vehicle index and not vehicle types.

### Compact formulation with loading variables

A main disadvantage of model F2 presented in Sect. 2.2 is that capacity constraints are not explicitly defined, requiring cuts to be added dynamically. This might lead to weak bounds at the early stages of its optimization. To overcome this situation, formulation F3 proposed in this section makes use of stronger constraints to handle capacity restrictions. We define additional continuous variables to help control the load of the vehicles. This model is based on the commodity flow formulation proposed by Garvin et al. (1957) for an oil delivery problem and later extended by Gavish and Graves (1982) to VRP variants. A similar formulation for the single depot VRP is given in Toth and Vigo (2002). Later, Baldacci et al. (2008) extended this formulation for the VRP with heterogeneous fleet, Salhi and Rand (1993) and Yaman (2006) extended it for the FSMVRP, Salhi et al. (2014) modified it to handle a VRP with multiple depots, and Koç et al. (2016a) amended it for the fleet size and mix location-routing problem with time windows. The formulation proposed in this section is quite different from the one proposed in Salhi et al. (2014) for the MDFSMVRP as we define new assignment variables $y_{i}^{{kd}}$ in addition to ${x_{i j}^{{kd}}}$. Indeed, Bosch and Trick (2005) highlight that adding variables and/or constraints to a formulation may strengthen the linear relaxation and provide improved formulations. They also state that for many problems, the use of integer variables, even when it is not required, may expand the capability of the model and help find an optimal solution.

The formulation is derived by defining new continuous variables $z_{{ij}}$ representing the remaining load on the vehicle when traversing arc (*i*, *j*), i.e., after visiting node *i* and before visiting node *j*. Note that the loading variables could be defined only for the asymmetric version of the problem since the complete graph is considered. In this graph, a vehicle must return to the depot empty. We also use the same four-index binary variables $x_{{ij}}^{{kd}}$ and the visiting binary variables $y_{i}^{{kd}}$ defined in Sect. 2.2 but on a directed graph. Indeed, in this model, (*i*, *j*) belongs to the arc set ${\mathcal {A}}$ and $x_{{ij}}^{{kd}}$ takes value one if arc $(i,j) \in {\mathcal {A}}$ traversed by vehicle type *k* housed at depot *d* is used in the solution, and zero otherwise. In what follows, we restate all the constraints of the problem dealing with routing variables $x_{i j}^{{kd}}$, since they are expressed differently from the constraints defined in models F1 and F2, despite having the same role. The formulation is defined by:

26 $$\begin{aligned} {\text {(F3) minimize}} \sum _{i\in {\mathcal {V}}_c} \sum _{k\in {\mathcal {K}}} \sum _{d\in {\mathcal {V}}_d} F^k {x_{di}^{{kd}}} + \sum _{(i,j)\in {\mathcal {A}}} \sum _{k\in {\mathcal {K}}} \sum _{d\in {\mathcal {V}}_d} {\alpha ^k \beta _{{ij}}}{x_{i j}^{{kd}}} \end{aligned}$$

subject to (2), (4) and to:

27 $$\begin{aligned} \sum _{j\in {\mathcal {V}}} x_{i j}^{{kd}} + \sum _{j\in {\mathcal {V}}} x_{j i}^{{kd}}= & {} 2y_{i}^{{kd}} \quad i\in {\mathcal {V}}_c, k\in {\mathcal {K}}, d\in {\mathcal {V}}_d \end{aligned}$$

28 $$\begin{aligned} \sum _{i\in {\mathcal {V}}} {x_{{ij}}^{{kd}}}= & {} \sum _{i\in {\mathcal {V}}} {x_{{ji}}^{{kd}}} \quad j\in {\mathcal {V}}, k\in {\mathcal {K}}, d\in {\mathcal {V}}_d \end{aligned}$$

29 $$\begin{aligned} y_{d}^{{kd}}\le & {} \sum _{(i,j)\in {\mathcal {A}}} x_{i j}^{{kd}} \quad k\in {\mathcal {K}}, d\in {\mathcal {V}}_d \end{aligned}$$

30 $$\begin{aligned} 2y_{d}^{{kd}}\le & {} \sum _{j\in {\mathcal {V}}_c} x_{jd}^{{kd}} + \sum _{j\in {\mathcal {V}}_c} x_{dj}^{{kd}}\quad k\in {\mathcal {K}}, d\in {\mathcal {V}}_d \end{aligned}$$

31 $$\begin{aligned} \sum _{i\in {\mathcal {V}}} z_{{ij}} - \sum _{i\in {\mathcal {V}}} z_{{ji}}= & {} q_j \quad j \in {\mathcal {V}}_c \end{aligned}$$

32 $$\begin{aligned} \sum _{i\in {\mathcal {V}}_d} \sum _{j\in {\mathcal {V}}_c} z_{{ij}}= & {} \sum _{j\in {\mathcal {V}}_c} q_j \end{aligned}$$

33 $$\begin{aligned} z_{{ij}}\le & {} \sum _{k\in {\mathcal {K}}} \sum _{d\in {\mathcal {V}}_d} (Q^k -q_i) x_{i j}^{{kd}} \quad i \in {\mathcal {V}}, j \in {\mathcal {V}}_c \end{aligned}$$

34 $$\begin{aligned}&x_{i j}^{{kd}} \in \{0,1\} \quad (i,j)\in {\mathcal {A}}, k\in {\mathcal {K}}, d\in {\mathcal {V}}_d \end{aligned}$$

35 $$\begin{aligned}&y_{i}^{{kd}} \in \{0,1\} \quad i\in {\mathcal {V}}, k\in {\mathcal {K}}, d\in {\mathcal {V}}_d \end{aligned}$$

36 $$\begin{aligned}&z_{{ij}} \ge 0 \quad i, j\in {\mathcal {V}}. \end{aligned}$$

The objective function (26) minimizes the total routing costs. Equations (2) enforce that each customer must be visited exactly once. Constraints (27) and (28) replace the flow conservation constraints (3) defined in model F1. Constraints (4), (29) and (30) are equivalent to constraints (4)–(6) in model F1. They enforce that only used vehicles may serve customers. Constraints (31)–(33) are specific to the commodity flow formulation. They impose both the connectivity of the solution and the vehicle capacity constraints. In particular, constraints (31) guarantee that each customer demand is satisfied. Summing up these constraints yields constraint (32) which states that the total load leaving all depots must be equal to the total customer demands. Constraints (33) bound the load on each arc (*i*, *j*), i.e., after visiting node *i* the load on arc (*i*, *j*) plus the demand of node *i* cannot exceed the capacity of the vehicle used. Constraints (34)–(36) define the domain and nature of the variables. Formulation F3 has the advantage that connectivity constraints are polynomial in size, unlike models F1 and F2 which require a branch-and-cut algorithm to dynamically add subtour elimination constraints which are exponential in number.

Because of the way new variables $z_{{ij}}$ are defined, it is possible to further tighten this formulation. We introduce bounding constraints as in Salhi et al. (2014). Constraints (37) impose a lower bound on loading variables. They state that the total load of arc (*i*, *j*) must be at least equal to the demand of node *i*.

37 $$\begin{aligned} z_{{ij}} \ge \sum _{k\in {\mathcal {K}}} \sum _{d\in {\mathcal {V}}_d} q_j x_{i j}^{{kd}} \quad i \in {\mathcal {V}}_c, j \in {\mathcal {V}}_c \end{aligned}$$

Constraints (38) and (39) enhance the flow conservation of the problem by imposing that the total flow entering a node must equal to the total flow leaving the node. Karaoglan et al. (2012) have introduced several classes of valid inequalities for the location-routing problem with simultaneous pick-up and delivery. Some of these constraints have been extended to the fleet size and mix location-routing problem with time windows in Koç et al. (2016a). We adapt these constraints in (40)–(42) to the MDFSMVRP. They exclude illegal vehicle routes that do not start and end at the same depot. Constraints (43) represent a special case of subtour elimination constraints on two-node sets. Constraints (44) bound the number of vehicles trips.

38 $$\begin{aligned} \sum _{i\in {\mathcal {V}}} \sum _{k\in {\mathcal {K}}} \sum _{d\in {\mathcal {V}}_d} {x_{i j}^{{kd}}}= & {} 1 \quad j\in {\mathcal {V}}_c \end{aligned}$$

39 $$\begin{aligned} \sum _{j\in {\mathcal {V}}} \sum _{k\in {\mathcal {K}}} \sum _{d\in {\mathcal {V}}_d} {x_{i j}^{{kd}}}= & {} 1 \quad i\in {\mathcal {V}}_c \end{aligned}$$

40 $$\begin{aligned} \sum _{k\in {\mathcal {K}}} {x_{id}^{{kd}}}\le & {} \sum _{k\in {\mathcal {K}}} {y_{i}^{{kd}}} \quad i\in {\mathcal {V}}_c, d\in {\mathcal {V}}_d \end{aligned}$$

41 $$\begin{aligned} \sum _{k\in {\mathcal {K}}} {x_{di}^{{kd}}}\le & {} \sum _{k\in {\mathcal {K}}} {y_{i}^{{kd}}} \quad i\in {\mathcal {V}}_c, d\in {\mathcal {V}}_d \end{aligned}$$

42 $$\begin{aligned} \sum _{k\in {\mathcal {K}}} {x_{{ij}}^{{kd}}} + \sum _{k\in {\mathcal {K}}} {y_{i}^{{kd}}} + \sum _{k\in {\mathcal {K}}} \sum _{h\in {\mathcal {V}}_d, h\ne d} {y_{j}^{kh}}\le & {} 2 \quad i,j\in {\mathcal {V}}_c, i\ne j, d\in {\mathcal {V}}_d \end{aligned}$$

43 $$\begin{aligned} x_{{ij}}^{{kd}} + x_{{ji}}^{{kd}}\le & {} 1 \quad i,j\in {\mathcal {V}}_c, k\in {\mathcal {K}}, d\in {\mathcal {V}}_d \end{aligned}$$

44 $$\begin{aligned} \left\lceil \frac{\sum _{i\in {\mathcal {V}}_c} q_i }{\max \{Q^k\}} \right\rceil\le & {} \sum _{i\in {\mathcal {V}}_c} \sum _{k\in {\mathcal {K}}} \sum _{d\in {\mathcal {V}}_d} x_{di}^{{kd}}. \end{aligned}$$

### Compact formulation with disaggregated loading variables

In this section, we propose a more detailed formulation based on F3 for the MDFSMVRP, referred to as F4. The motivation is to carry information related to the vehicle type on each arc by disaggregating the loading variables $z_{{ij}}$. We define new continuous variables $z_{{ij}}^k$, such that $z_{{ij}}= \sum _{k\in {\mathcal {K}}} z_{{ij}}^k$. This model is inspired from the work of Yaman (2006) for the FSMVRP. The model is defined by minimizing (26) subject to (2), (4), (27)–(30), (34), (35) and to:

45 $$\begin{aligned} \sum _{i\in {\mathcal {V}}_d} \sum _{j\in {\mathcal {V}}_c} \sum _{k\in {\mathcal {K}}} z_{{ij}}^k= & {} \sum _{j\in {\mathcal {V}}_c} q_j \end{aligned}$$

46 $$\begin{aligned} \sum _{i\in {\mathcal {V}}} z_{{ij}}^k - \sum _{i\in {\mathcal {V}}} z_{{ji}}^k= & {} \sum _{d\in {\mathcal {V}}_d} q_j y_{j}^{{kd}} \quad j \in {\mathcal {V}}_c, k\in {\mathcal {K}} \end{aligned}$$

47 $$\begin{aligned} z_{{ij}}^k\le & {} \sum _{d\in {\mathcal {V}}_d} \left( Q^k -q_i\right) x_{i j}^{{kd}} \quad i \in {\mathcal {V}}, j \in {\mathcal {V}}_c, k\in {\mathcal {K}} \end{aligned}$$

48 $$\begin{aligned} z_{{ij}}^k\ge & {} 0 \quad i, j\in {\mathcal {V}}, k\in {\mathcal {K}}. \end{aligned}$$

Constraints (45)–(47) have a similar meaning as constraints (31)–(33) of model F3. The only exception is that they provide more precision on the vehicle type carrying the load on arc (*i*, *j*). Formulation F4 has more continuous variables and constraints compared to F3.

Model F4 can also be strengthened by (38)–(44), while constraints (37) must be replaced by (49):

49 $$\begin{aligned} z_{{ij}}^k \ge \sum _{d\in {\mathcal {V}}_d} q_{j} x_{{ij}}^{{kd}} \quad i \in {\mathcal {V}}_c, j \in {\mathcal {V}}_c, k\in {\mathcal {K}}. \end{aligned}$$

### Capacity-indexed formulation

In this section, we make use of a novel formulation to model VRPs, referred to as capacity-indexed formulation. This type of formulation has only appeared a few times for basic variants of VRPs. A seminal paper proposing a capacity-indexed formulation for the time-dependent traveling salesman problem is due to Picard and Queyranne (1978). Godinho et al. (2008) used it for the case of unitary demands. Later, Pessoa et al. (2008) and Poggi de Aragão and Uchoa (2014) propose a similar formulation for the asymmetric VRP, and Pessoa et al. (2007) and Pessoa et al. (2009) extend this model to handle the asymmetric VRP with heterogeneous fleet.

We define new binary variables $x_{{ij}}^{{kdq}}$ equal to one if and only if vehicle type *k* housed at depot *d* traverses arc (*i*, *j*) with a load of *q* units. This variable indicates the load of a given vehicle type housed at a given depot on a given arc, unlike the commodity flow formulations (F3 and F4) that require the definition of continuous variables to convey similar information. Vehicles returning to the depot must have a load *q* equal to zero and a vehicle *k* traversing arc (*i*, *j*) must not carry a load *q* lower that the demand of node *j*. This model can then be formulated as follows:

50 $$\begin{aligned} {\text {(F5) minimize}} \sum _{i\in {\mathcal {V}}_c} \sum _{k\in {\mathcal {K}}} \sum _{d\in {\mathcal {V}}_d} \sum _{q=1}^{{Q}^k} F^k {x_{di}^{{kdq}}} + \sum _{(i,j)\in {\mathcal {A}}} \sum _{k\in {\mathcal {K}}} \sum _{d\in {\mathcal {V}}_d} \sum _{q=0}^{{Q}^k} {\alpha ^k \beta _{{ij}}}{x_{i j}^{{kdq}}} \end{aligned}$$

subject to

51 $$\begin{aligned}&\sum _{j\in {\mathcal {V}}} \sum _{k\in {\mathcal {K}}} \sum _{d\in {\mathcal {V}}_d} \sum _{q=1}^{{Q}^k} {x_{{ji}}^{{kdq}}} =1 \quad i\in {\mathcal {V}}_c \end{aligned}$$

52 $$\begin{aligned}&\sum _{i\in {\mathcal {V}}_c} \sum _{q=1}^{{Q}^k} {x_{di}^{{kdq}}} =\sum _{i\in {\mathcal {V}}_c} {x_{id}^{kd0}} \quad k\in {\mathcal {K}}, d\in {\mathcal {V}}_d \end{aligned}$$

53 $$\begin{aligned}&\sum _{j\in {\mathcal {V}}} {x_{{ji}}^{{kdq}}} =\sum _{j\in {\mathcal {V}}} {x_{{ij}}^{kd (q-q_i)}} \quad i\in {\mathcal {V}}_c, k\in {\mathcal {K}}, d\in {\mathcal {V}}_d, q=\{q_i, \ldots , Q^k\} \end{aligned}$$

54 $$\begin{aligned}&x_{i j}^{{kdq}} \in \{0,1\} \quad i,j\in {\mathcal {V}}, k\in {\mathcal {K}}, d\in {\mathcal {V}}_d, q=\{0, \ldots , Q^k\}. \end{aligned}$$

The total routing costs are minimized in (50). Equation (51) consists of in-degree constraints. They ensure that each customer is visited exactly once. Constraints (52) ensure flow conservation and guarantee that if a vehicle of type *k* leaves a depot *d* with a load *q* then it must return to this depot with a load equal to 0. The connectivity of the solution and the vehicle capacity requirements are ensured due to constraints (53). If vehicle *k* carrying a load $$q_i\le q\le Q^k$$ enters a node *i*, then it must leave it with a load equal to $$q-q_i$$. Constraints (54) define the domain of the capacity-indexed variables. In order to reduce the research space when using capacity-indexed variables, one can further remove unnecessary variables. We eliminate variables related to vehicle *k* traversing an arc (*i*, *j*) with an irrelevant load, i.e., after visiting a node *i*, the vehicle should not carry a load between $$Q^k - q_i $$ and the capacity of vehicle *k*, $$Q^k$$. Those unnecessary variables are removed with equalities (55):

55 $$\begin{aligned} x_{{ij}}^{{kdq}}=0 \quad i\in {\mathcal {V}}, j\in {\mathcal {V}}_c, k\in {\mathcal {K}}, d\in {\mathcal {V}}_d, Q^k - q_i <q \le Q^k. \end{aligned}$$

Solving the MDFSMVRP directly with this formulation is practical only for small values of $Q^k$. We derive new valid inequalities in the form of balance and capacity constraints and routing constraints, which impose bounds on the binary variables. Constraints (56) and (57) are inspired from those proposed in Pessoa et al. (2009). They impose a lower bound on the number of vehicle routes and the number of variables, respectively. Equation (58) consists of balance constraints. They state that if vehicle *k* traversing arc (*i*, *j*) enters node *i*, then the load delivered to node *i* must be exactly $q_i$.

56 $$\begin{aligned}&\left\lceil \frac{\sum _{i\in {\mathcal {V}}_c} q_i }{\max \{Q^k\}} \right\rceil \le \sum _{i\in {\mathcal {V}}_c} \sum _{k\in {\mathcal {K}}} \sum _{d\in {\mathcal {V}}_d} \sum _{q=q_i}^{{Q}^k} x_{di}^{{kdq}} \end{aligned}$$

57 $$\begin{aligned}&\sum _{i\in {\mathcal {V}}_c} q_i \le \sum _{i\in {\mathcal {V}}} \sum _{j\in {\mathcal {V}}_c} \sum _{k\in {\mathcal {K}}} \sum _{d\in {\mathcal {V}}_d} \sum _{q=q_i}^{{Q}^k} qx_{{ij}}^{{kdq}} \end{aligned}$$

58 $$\begin{aligned}&\sum _{j\in {\mathcal {V}}} \sum _{k\in {\mathcal {K}}} \sum _{d\in {\mathcal {V}}_d} \sum _{q=q_i}^{{Q}^k-q_j} q x_{{ji}}^{{kdq}} - \sum _{j\in {\mathcal {V}}} \sum _{k\in {\mathcal {K}}} \sum _{d\in {\mathcal {V}}_d} \sum _{q=q_j}^{{Q}^k-q_i} q x_{{ij}}^{{kdq}} = q_i \quad i\in {\mathcal {V}}_c. \end{aligned}$$

Constraints (59)–(61) are referred to as routing constraints, as a way to ensure that if there is an arc (*i*, *j*) related to vehicle *k* and linking two customers *i* and *j*, i.e., (59) holds, then there must be at least one arc traversed by *k* and returning to depot *d*, i.e., (60) holds. Equalities (61) are outgoing arcs, and they reinforce equation (51).

59 $$\begin{aligned}&\sum _{q=q_j}^{{Q}^k-q_i} x_{{ij}}^{{kdq}} \le \sum _{h\in {\mathcal {V}}_c} x_{hd}^{kd0}\quad i,j\in {\mathcal {V}}_c, k\in {\mathcal {K}}, d\in {\mathcal {V}}_d \end{aligned}$$

60 $$\begin{aligned}&x_{hd}^{kd0} \le \sum _{i\in {\mathcal {V}}} \sum _{j\in {\mathcal {V}}_c} \sum _{q=q_j}^{{Q}^k -q_i} x_{{ij}}^{{kdq}} \quad h\in {\mathcal {V}}_c, k\in {\mathcal {K}}, d\in {\mathcal {V}}_d \end{aligned}$$

61 $$\begin{aligned}&\sum _{j\in {\mathcal {V}}} \sum _{k\in {\mathcal {K}}} \sum _{d\in {\mathcal {V}}_d} \sum _{q=0}^{{Q}^k} {x_{{ij}}^{{kdq}}} =1 \quad i\in {\mathcal {V}}_c. \end{aligned}$$

## Theoretical insights

A large number of formulations have been proposed for the VRP. However, a much smaller number of papers have discussed the theoretical formulations of these models, comparing their structures and dominance (Letchford and Salazar-González 2006). Many researchers have focused on studying the polyhedral structure of one particular model at a time (Laporte and Nobert 1984; Araque et al. 1990; Campos et al. 1991; Cornuéjols and Harche 1993; Augerat 1995; Gouveia 1995b; Letchford et al. 2002; Godinho et al. 2008; Gouveia and Salazar-González 2013; Gouveia et al. 2013; Bektaş and Gouveia 2014). Very few compare different formulations, e.g., Gouveia (1995b), Letchford and Salazar-González (2006) and Yaman (2006). In what follows we discuss their main results and relations to the models presented in Sect. 2.

The seminal work of Gouveia (1995b) describes the set of feasible solutions of a one-commodity flow formulation for the one-unit VRP (Gavish and Graves 1982), comparing the polyhedral structure against existing weaker variants of that formulation. Based on these results, the author proposed a new and better extended formulation for the problem, besides generalizing the results for VRPs with non-unit demands. These families of commodity flow formulations use two sets of variables: routing variables to describe the design of the routes, and another set of continuous loading variables to capture the load of the vehicle traversing each arc. The load variables are then used to enforce capacity and subtour elimination constraints. This idea is exploited in formulations F3 and F4 proposed in Sects. 2.3 and 2.4. Gouveia (1995b) stated that flow-based formulations used for capacitated problems can lead to good lower bounds, and yield interesting results when used in combinations with some families of valid inequalities. This has been successfully used to solve the capacitated minimal spanning tree problem (Gouveia 1995a), the VRP (Toth and Vigo 2002) and the FSMVRP (Yaman 2006). Moreover, Gouveia (1995b) stated that the commodity flow formulation using loading variables to enforce subtour and capacity constraints, such as (31)–(33), performs better than models exploiting binary variables and generalized subtour elimination constraints of the form (25). The information regarding load variables leads to a more compact formulation, with fewer constraints that are easier to lift. The author demonstrated that result by analyzing the projection of the commodity flow formulation into the two-index vehicle flow space. Toth and Vigo (2002) have later revalidated the same results. The authors have stated that two- and three-index vehicle flow formulations using binary routing variables to enforce capacity and subtour elimination constraints, i.e., the models on which formulations F1 and F2 of Sects. 2.1 and 2.2 are based, cannot be used efficiently to solve problems with some operational constraints, such as those with a fixed vehicle cost depending on its type. The authors have also stated that the linear programming (LP) relaxation of these two models is very weak.

Letchford and Salazar-González (2006) have later proposed the commodity flow formulation as an alternative for the two- and three-index vehicle flow formulations, based on the fact that these latter require an exponential number of constraints to enforce connectivity. Moreover, they have shown that the integer relaxation of the commodity flow model is generally not dominated by that of the vehicle flow models. Gouveia (1995b) had suggested using the linear relaxation of the commodity flow formulation strengthened by inequalities (33) when starting a branch-and-cut procedure. However, since the number of (33) is exponential in *n*, the author points out that the size of the linear programming problem may become an issue.

This latter disadvantage may be overcome by using a more compact model replacing binary routing variables by binary ones indicating the flow on each arc (Gouveia 1995b). By doing this, one replaces $O(n^2)$ constraints of type (33) by *O*(*n*) constraints of type (53). This is the idea exploited by model F5 presented in Sect. 2.5. Recently, Yaman (2006) have performed a deep theoretical analysis of several different formulations and valid cuts for the heterogeneous VRP. She has compared LP bounds of flow formulations and Miller–Tucker–Zemlin (MTZ) formulations through projection. The main result indicates that the LP bounds of formulations with capacity and subtour elimination constraints modeled with MTZ constraints (Miller et al. 1960) and binary routing variables give raise to a weak estimate of the optimal value compared to the LP bounds of the formulations using flow variables to express the capacity and the subtour elimination constraints. The author has also shown that the LP relaxation of the formulations with disaggregating flow variables outperforms that of the original aggregated flow formulation, which in turn outperforms the LP relaxation of vehicle flow formulations.

In Sect. 5, we provide a comparison of the performance of the five proposed models from an empirical perspective. As has been the choice of several other researchers, we will analyze and discuss the results of the LP relaxation, the bounds obtained, and the efficiency of some families of the proposed valid inequalities with a focus on the compact formulations. All tests will be performed on various publicly available instances.

## Solution algorithms

The formulations presented in Sects. 2.3, 2.4, and 2.5 can be explicitly generated and one can enumerate all variables and constraints. These can then be fed into a general purpose solver and solutions are obtained by branch-and-bound. However, the models presented in Sects. 2.1 and 2.2 cannot be fully generated due to constraints (7) and (25) which are in the order of $O(2^n)$. Thus, one needs to dynamically generate them only if they are found to be violated in a partial solution. The exact algorithm we present is then a classical branch-and-cut which works as follows. At a generic node of the search tree, a linear program including a subset of the subtour elimination constraints is solved, a search for violated constraints is performed, appropriate valid inequalities are added to eliminate subtours, and the current subproblem is then reoptimized. This process is reiterated until a feasible or dominated solution is reached, or until no more cuts can be added. At this point, branching on a fractional variable occurs. We provide a sketch of the branch-and-cut scheme in Algorithm 1.
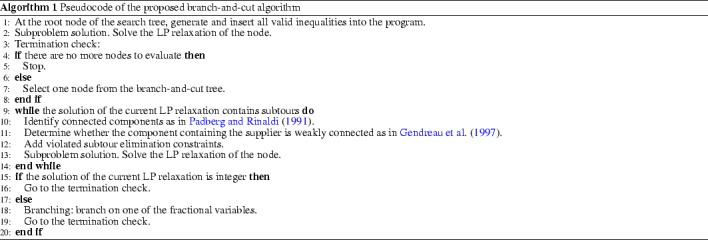


Finally, the model presented in Sect. 2.5 can be fully enumerated for most small and medium size instances. However, it is easy to observe that some variables are never used in the model, e.g., those for which values of *q* cannot be obtained by any combination of demands. These variables can be generated and fed to the solver, which will set them to zero in any feasible solution. If one can identify these variables beforehand, it is possible to set them to zero and remove then from the model at a preprocessing phase. Thus, one can (substantially) decrease the size of the model and the memory usage by preprocessing the model and the instance a priori, identifying the subset of variables that should not be generated. We have then implemented a subset sum algorithm to identify all possible values of *q* from 1 to ${Q}^k$ that can be achieved by any combination of demands $q_i$. The ones that are found not to be feasible are not generated and we could substantially reduce the size of the model. Details regarding the improvements provided by this algorithm are presented in Sect. 5.3.

## Computational experiments

In this section we provide details on the implementation, benchmark instances, and describe the computational experiments we have performed. Implementation and hardware information is provided in Sect. 5.1. The description of the existing and new benchmark instances we have used are presented in Sect. 5.2, followed by the results of our extensive computational experiments in Sect. 5.3.

### Implementation details

All the formulations described in Sect. 2 were implemented in C++ and solved with IBM CPLEX Concert Technology 12.5.1. We have used the nearest neighbor heuristic to provide the solver with a trivial initial solution. To better exploit our resources, we allow CPLEX to invoke up to 12 parallel threads and to run for 3 h on each execution. All other parameters are kept to a default setting, as our tests have shown no significant gains could be obtained. The separation of the subtour elimination constraints was performed with the Concorde package of Applegate et al. (2011) and the CVRPSEP package of Lysgaard et al. (2004).

We have run all instances described in the next section using all models described in Sect. 2 with a time limit of 3 h and a maximum of 96 Gb of memory. The machines used are all equipped with Intel Xeon™ processors running at 2.66 GHz with 96 GB of RAM installed per node, with the Scientific Linux 6.1 operating system.

### Description of the instances

In order to compare the performance of our models and algorithms, we have used a set of 14 test instances proposed by Salhi and Sari (1997) for the MDFSMVRP. These instances were inspired from older benchmarks for other vehicle routing problems proposed by Gillett and Johnson (1976), Perl and Daskin (1985) and Chao et al. (1993). They are commonly used in the VRP literature. They have been used in previous researches to evaluate the performance of heuristic algorithms, namely the multi-level composite heuristic of Salhi and Sari (1997), the variable neighborhood search of Salhi et al. (2014), and the hybrid genetic search with advanced diversity control of Vidal et al. (2014). The only lower bounds and solutions obtained with an exact approach existing for these instances were obtained by a branch-and-bound algorithm applied to a mathematical model presented in Salhi et al. (2014).

These instances contain between 50 and 360 customers, and between two and nine depots. There are five vehicle types, i.e., $K=5$, in all instances. The vehicle capacities are generated centered around the value of the vehicle capacity (${\hat{Q}}$) of the original instances designed for of the MDVRP data sets. The vehicle capacities ${Q}^k$ along with the vehicle variable cost $F^k$ and the vehicle fixed cost $\alpha ^k$ are derived based on the following formulas: $Q^k=(0.4+0.2k){\hat{Q}}$, $F^k=70+10k$ and $\alpha ^k=0.7+0.1k$, with $k=1,\ldots ,5$.

We have also generated ten smaller instances to better evaluate the performance of the different formulations in terms of lower and upper bounds, and of running times. These instances were created by randomly selecting subsets of customers from the smaller instances of Salhi and Sari (1997), namely instances 4-55-100 and 4-50-80. Our instances contain two and three depots, from 10 to 30 customers, five vehicle types, and different demands distribution. Table 1 contains a list of all instances used in this paper and provides additional information on their origins and sizes. An asterisk is used to distinguish between the newly generated instances having the same number of customers, depots and vehicle capacity, but different demands and node location.

**Table 1:** Configurations of the existing and newly generated instances

Instance	References	Origin	# Depots	# Customers	${\hat{Q}}$
4-55-100	Salhi and Sari (1997)	Perl and Daskin (1985)	4	55	100
3-85-100	Salhi and Sari (1997)	Perl and Daskin (1985)	3	85	100
3-85-160	Salhi and Sari (1997)	Perl and Daskin (1985)	3	85	160
4-50-80	Salhi and Sari (1997)	Gillett and Johnson (1976)	4	50	80
4-50-160	Salhi and Sari (1997)	Gillett and Johnson (1976)	4	50	160
5-75-140	Salhi and Sari (1997)	Gillett and Johnson (1976)	5	75	140
2-100-100	Salhi and Sari (1997)	Gillett and Johnson (1976)	2	100	100
2-100-200	Salhi and Sari (1997)	Gillett and Johnson (1976)	2	100	200
3-100-100	Salhi and Sari (1997)	Gillett and Johnson (1976)	3	100	100
4-100-100	Salhi and Sari (1997)	Gillett and Johnson (1976)	4	100	100
2-80-60	Salhi and Sari (1997)	Chao et al. (1993)	2	80	60
4-160-60	Salhi and Sari (1997)	Chao et al. (1993)	4	160	60
6-240-60	Salhi and Sari (1997)	Chao et al. (1993)	6	240	60
9-360-60	Salhi and Sari (1997)	Chao et al. (1993)	9	360	60
2-10-60	New	Salhi and Sari (1997)	2	10	60
2-15-60	New	Salhi and Sari (1997)	2	15	60
3-20-80	New	Salhi and Sari (1997)	3	20	80
3-25-80	New	Salhi and Sari (1997)	3	25	80
3-30-80	New	Salhi and Sari (1997)	3	30	80
2-10-60*	New	Salhi and Sari (1997)	2	10	60
2-15-60*	New	Salhi and Sari (1997)	2	15	60
3-20-100	New	Salhi and Sari (1997)	3	20	100
3-25-100	New	Salhi and Sari (1997)	3	25	100
3-30-100	New	Salhi and Sari (1997)	3	30	100

### Computational experiments

In this section, we describe the results of computational experiments carried out in order to assess the performance of the proposed models and algorithms. Table 2 recalls the configurations of the five formulations tested and their origins.

**Table 2:** Summary of the five formulations

#	Formulation name	Origin	O.F. and constraints
F1	Explicit vehicle index formulation	Vidal et al. (2014) with introducing new assignment variables $y_{i}^{{td}}$	(1) s.t. (2) –(23)
F2	Implicit vehicle index formulation	F1 with vehicle index denoting vehicle type instead of individual vehicles	(24) s.t. (2)–(6), (7), (9)–(11), (15)–(21), (25)
F3	Compact formulation with loading variables	Salhi et al. (2014) with introducing new assignment variables $y_{i}^{{kd}}$	(26) s.t. (2), (4), (27)–(44)
F4	Compact formulation with disaggregated loading variables	F3 with disaggregating the continuous loading variables $z_{{ij}}$	(26) s.t. (2), (4), (27)–(30), (34), (35), (38)–(49)
F5	Capacity-indexed formulation	Pessoa et al. (2009)	(50) s.t. (51)–(61)

As stated in Sect. 4, Formulation F5 can be defined only for the values of *q* that can be attained, which can significantly reduce its size. For the existing instances described in Table 1, the average number of variables of F5 is reduced from 22,395,311 to 19,636,711 when applying the preprocessing step with the subset sum algorithm. We note that for the largest instance of the testbed, 9-360-60, which contains nine depots and 360 customers, the number of required variables could not be enumerated due to memory usage (it required more than 100 Gb of RAM memory). We also observe that the efficiency of the preprocessing phase is highly dependent on the scale and the distribution of the demands. For example, if all demands are multiples of 20, then the number of variables is reduced by almost 20-fold; however, if they are all small and some are unitary, then almost all values of $Q^k$ can be obtained by combining the demands of some customers. In this testbed, the number of generated variables is reduced by more than 16 times for instance 4-55-100, while it remains unchanged for 2-100-100. These values can be observed for all instances in Table 3.

**Table 3:** Number of generated variables for model F5 before and after the preprocessing phase

Instance	Before preprocessing	After preprocessing
4-55-100	7,031,620	417,720
3-85-100	11,732,160	696,960
3-85-160	18,701,760	998,976
4-50-80	4,723,920	4548,960
4-50-160	9,389,520	9,214,560
5-75-140	22,560,000	22,400,000
2-100-100	10,508,040	10,508,040
2-100-200	20,912,040	20,912,040
3-100-100	16,072,635	16,072,635
4-100-100	21,848,320	21,848,320
2-80-60	4,101,640	4,101,640
4-160-60	32,813,120	32,813,120
6-240-60	110,744,280	110,744,280
9-360-60	Out of memory	Out of memory
Average	22,395,311.92	19,636,711.62

#### Linear programming relaxation

Solving the linear programming relaxation (LR) can be quite useful as it provides a bound on the optimal value of the integer programs, and it highlights the difference between the formulations. The first experiment we conduct in this section consists of solving the LRs of the five formulations for both data sets with a time limit of 2 h. We include all the valid inequalities presented in the previous sections. Table 4 summarizes the results of this test. For each model and each solved instance, we provide the LR value and the running time in seconds. In all tables, if an instance cannot be solved, we mention NF indicating not found status, and NC if the number of required variables and constraints could not be enumerated. Boldface is used to indicate the new best results. The results indicate that the LR of model F1 is quite poor. This is due to the drawbacks of this formulation mentioned before, particularly the fact of enumerating the available vehicles. Formulation F1 does not provide a solution to the linear relaxation within 2 h for any instance of the first data set. Furthermore, comparing the last four formulations substantiates that models F3 and F4 perform extremely well on both data sets compared to model F2. The average of the LR values, only over solved instances (from instance 4-55-100 to 2-80-60), equals 790.94, 1554.14, and 1649.38 while the average running time is increasing from 55 to 77 and to 574 s for models F2, F3, and F4, respectively. The average computation time of model F4 is almost seven times the average computation time of model F3, whereas the difference between the LRs of these two formulations is small. This implies that disaggregating loading variables requires more computational time to find slightly better relaxations. Model F5 provides better LR values for all 15 solved instances compared to all other formulations. Over all models, the average time taken to solve the LRs is not negligible. This is due to the high number of variables and constraints required to model the problem.

**Table 4:** Linear programming relaxations for the five formulations

Instance	Formulation F1		Formulation F2		Formulation F3		Formulation F4		Formulation F5	
	Value	Time (s)	Value	Time (s)	Value	Time (s)	Value	Time (s)	Value	Time (s)
4-55-100	NF	7200	574.32	17	1313.54	29	1354.41	58	**1359**.**99**	56
3-85-100	NF	7200	841.57	43	2027.98	66	2079.01	456	**2094**.**42**	157
3-85-160	NF	7200	631.65	44	1347.39	77	1411.31	505	**1435**.**19**	357
4-50-80	NF	7200	642.90	23	1322.30	21	1381.63	58	**1416**.**05**	4348
4-50-160	NF	7200	496.07	10	807.47	17	**890**.**99**	89	NF	7200
5-75-140	NC	7200	707.52	62	1362.47	102	**1478**.**41**	530	NF	7200
2-100-100	NC	7200	966.10	53	2095.84	94	**2191**.**73**	816	NF	7200
2-100-200	NC	7200	749.49	54	1262.37	80	**1382**.**34**	1021	NF	7200
3-100-100	NC	7200	952.52	84	1990.05	143	**2106**.**65**	1074	NF	7200
4-100-100	NC	7200	951.76	166	1993.18	197	**2103**.**29**	1422	NF	7200
2-80-60	NC	7200	1186.35	48	1573.03	24	1763.45	287	**1790**.**17**	6861
4-160-60	NC	7200	NF	7200	**3063**.**71**	1090	NF	7200	NF	7200
6-240-60	NC	7200	NF	7200	NF	7200	NF	7200	NF	7200
9-360-60	NC	7200	NF	7200	NF	7200	NF	7200	NC	7200
2-10-60	268.51	5	268.58	0	399.87	0	409.55	0	**422**.**33**	3
2-15-60	355.68	50	356.02	0	600.23	0	627.41	1	**650**.**23**	9
3-20-80	333.43	540	334.30	0	594.47	1	626.40	2	**640**.**66**	117
3-25-80	395.63	1880	397.93	1	706.59	1	744.38	6	**759**.**54**	218
3-30-80	NF	7200	438.25	2	828.87	3	866.25	7	**884**.**23**	393
2-10-60*	245.29	3	246.38	0	448.59	0	459.77	0	**468**.**59**	0
2-15-60*	316.37	43	318.53	0	646.36	0	659.91	1	**681**.**01**	0
3-20-100	255.55	206	255.41	1	525.82	1	544.05	2	**544**.**71**	2
3-25-100	303.23	1299	303.47	1	631.16	1	658.49	4	**660**.**80**	3
3-30-100	382.75	2216	383.20	1	784.34	2	820.20	9	**824**.**98**	5

#### Comparison of upper and lower bounds

We now present the computational results of the solutions we have obtained when applying branch-and-bound and branch-and-cut for the five proposed formulations. Table 5 summarizes the results after 3 h of running time with CPLEX. We report the upper bound (UB) and the lower bound (LB) of each formulation for each instance, if they are found. We provide the average percentage gaps over the two testbeds. The percentage gap is given by the ratio ($\frac{\hbox {UB}-\hbox {LB}}{\hbox {UB}}100$). We also give the average time in seconds spent to solve the new testbed. Bold face is used to indicate the best results.

A deeper analysis of the formulations highlights a remarkable improvement over all the lower bounds and the number of solved instances compared to the LRs results. The results clearly show that formulation F1 is outperformed by all the other formulations, even on small instances. The largest instance size that can be solved by formulation F1 is 4-5-160. Model F1 could identify a feasible solution only for three (out of 14) instances, whereas formulation F2 is able to solve all the instances of the two testbeds. This implies that the compact formulation, reducing the number of generated variables, has a positive impact on the model performance. Model F2 provides tighter bounds compared to F1 but is still uncompetitive compared to the other formulations. The results of Table 5 distinctly show the performance of the last three formulations to solve the MDFSMVRP. Model F4 could generally provide better bounds compared to all other formulations, especially on the first testbed, despite the fact that model F3 yields better UBs. We observe that there is a difference between models F3 and F4 regarding the overall gaps. The solutions provided by F4 are 8.2 and 1.2% better than the solutions provided by F3 on the two testbeds, respectively. Model F4 provides eight best LBs and five best UBs over 14 instances, while F3 provides eight best UBs on the first testbed. This implies that disaggregating the commodity flow variables is likely improving the model performance. Model F5 has better bounds on the first three instances compared to all other formulations and provides the best gap for instances 2-100-100 and 2-80-60. This is due to the fact that few variables are generated in these test instances, characterized by a regular distribution of customers demands and/or a small number of customers. However, even if model F5 provides six best LBs over 14 instances, its overall average gap is about three times the overall average gap of model F4 since seven instances out of 14 are not solved. Regarding the small generated instances, F5 outperforms all the other formulations and provides eight optimal solutions over 10, with an average gap equal to 0.57% and an average running time of 2390 s. F4 provides competitive solutions with slightly better average gap (0.49%) than F5 within less computation time (1832 s). However, formulation F4 proves the optimality only for the smallest instance with two depots and 10 customers. The computation times and the average gaps provided by formulations F1 and F2 on these small instances are quite high. In particular they require, on average, 8590 and 8841 s to solve instances with up to three depots and 30 customers.

These results point out again that formulations F3, F4 and F5 are the most suitable among the five proposed to solve small-, medium-, and large-size instances of the MDFSMVRP. Particularly, if the aim is to provide good upper bounds to compare with an heuristic, then it would be better to use formulation F3 on large instances and formulation F5 on small instances. Similarly, if major problems of running out of memory occur, one should use formulation F5 as fewer variables will be generated, especially with a regular distribution of customers demands. Finally, if the goal is to get a good trade-off between solution quality and running time, one should choose formulation F4 as disaggregating continuous variables is likely improving the model performance.

A transversal analysis over Tables 4 and 5 allows us to remark that the deductions derived after solving the models with integrality restrictions confirm the preliminary results derived from the LR experiment. This analysis also shows that the experimental results reassert the theoretical findings discussed in Sect. 3. In addition, we observe that, on average, the values of the LR of models F3 and F4 over the first 11 solved instances in Table 4 is equal to almost 0.7 and 0.8 times the UBs of the 3-h execution of these models. Thereby, one can conclude that the last three compact models proposed, especially the commodity flow formulations are good enough as they provide strong linear relaxations. Finally, we can derive some comments on the relative difficulty of the problem. We observe that the average gaps remain large, especially on instances with more than two depots and 100 customers.

**Table 5:** Summary of computational results obtained from the five formulations

Instance	Formulation F1			Formulation F2			Formulation F3			Formulation F4			Formulation F5		
	UB	LB	Gap (%)	UB	LB	Gap (%)	UB	LB	Gap (%)	UB	LB	Gap (%)	UB	LB	Gap (%)
4-55-100	1612.30	552.52	65.73	1606.63	575.62	64.17	1438.50	1328.99	7.61	1408.25	1361.85	3.29	**1398**.**30**	**1384**.**55**	0.98
3-85-100	NF	NF	–	2258.22	842.50	62.69	2209.95	2040.45	7.67	2163.63	2086.32	3.57	**2131**.**46**	**2116**.**12**	0.72
3-85-160	NF	NF	–	1698.58	633.22	62.72	1675.05	1360.58	18.77	1663.01	1423.30	14.41	**1483**.**51**	**1451**.**86**	2.13
4-50-80	1770.66	602.44	65.98	1770.66	621.48	64.90	1573.68	1348.51	14.31	**1565**.**27**	1390.51	11.16	1733.20	**1416**.**09**	18.30
4-50-160	1217.74	485.68	60.12	1216.52	499.13	58.97	**1021**.**59**	832.21	18.54	1122.08	**907**.**71**	19.10	1217.74	902.18	25.91
5-75-140	NC	NC	–	1962.67	708.06	63.92	1831.02	1388.41	24.15	**1828**.**73**	**1483**.**11**	18.92	NF	NF	–
2-100-100	NC	NC	–	2757.95	968.67	64.88	**2660**.**50**	2116.36	20.45	2725.87	2201.16	19.25	2757.95	**2236**.**91**	18.89
2-100-200	NC	NC	–	1826.28	750.27	58.92	**1818**.**69**	1280.61	29.59	1825.91	**1396**.**05**	23.54	NF	NF	–
3-100-100	NC	NC	–	2728.89	953.12	65.07	**2648**.**17**	2013.95	23.95	2696.05	**2109**.**31**	21.76	NF	NF	–
4-100-100	NC	NC	–	2691.82	952.05	64.63	**2626**.**33**	2003.95	23.70	2684.89	**2104**.**36**	21.63	NF	NF	–
2-80-60	NC	NC	–	2573.82	1186.35	53.91	2566.13	1683.51	34.39	**2565**.**53**	1788.35	30.29	2571.35	**1794**.**38**	30.22
4-160-60	NC	NC	–	5172.65	2199.05	57.49	**5157**.**49**	3094.10	40.01	5172.65	**3506**.**89**	32.20	NF	NF	–
6-240-60	NC	NC	–	7758.97	3182.68	58.98	**7758**.**97**	4564.18	41.18	**7758**.**97**	**5243**.**12**	32.43	NF	NF	–
9-360-60	NC	NC	–	11638.50	4441.25	61.84	**11638**.**50**	0.00	100.00	**11638**.**50**	**7852**.**44**	32.53	NC	NC	–
Average gap			90.99			61.65			28.88			20.63			56.94
2-10-60	**441**.**59**	**441**.**59**	0.00	**441**.**59**	441.57	0.00	**441**.**59**	**441**.**59**	0.00	**441**.**59**	**441**.**59**	0.00	**441**.**59**	**441**.**59**	0.00
2-15-60	679.18	611.92	9.90	679.18	489.20	27.97	**674**.**32**	674.26	0.01	**674**.**32**	674.26	0.01	**674**.**32**	**674**.**32**	0.00
3-20-80	784.97	354.03	54.90	757.34	405.73	46.43	**666**.**02**	665.95	0.01	**666**.**02**	665.97	0.01	**666**.**02**	**666**.**02**	0.00
3-25-80	845.41	409.43	51.57	839.13	429.99	48.76	794.46	745.08	6.21	**787**.**37**	787.29	0.01	**787**.**37**	**787**.**30**	0.01
3-30-80	1009.93	448.00	55.64	1005.05	448.83	55.34	932.13	870.77	6.58	932.13	**890.71**	4.44	**940**.**49**	886.95	5.69
2-10-60*	**482**.**09**	**482**.**09**	0.00	**482**.**09**	482.05	0.01	**482**.**09**	**482**.**09**	0.00	**482**.**09**	**482**.**09**	0.00	**482**.**09**	**482**.**09**	0.00
2-15-60*	**690**.**38**	**690**.**38**	0.00	**690**.**38**	393.98	42.93	**690**.**38**	690.31	0.01	**690**.**38**	690.31	0.01	**690**.**38**	**690**.**38**	0.00
3-20-100	597.44	543.12	9.09	605.95	293.85	51.51	**563**.**19**	563.13	0.01	**563**.**19**	563.15	0.01	**563**.**19**	**563**.**19**	0.00
3-25-100	727.54	651.16	10.50	717.86	321.37	55.23	**676**.**59**	674.68	0.28	**676**.**59**	676.53	0.01	**676**.**59**	**676**.**59**	0.00
3-30-100	994.17	735.29	26.04	994.17	397.68	60.00	**839**.**98**	812.71	3.25	**839**.**98**	836.77	0.38	**839**.**98**	**839**.**98**	0.00
Average gap/ time (s)			21.76/8590.10			38.82/8811.400			1.64/5138.40			0.49/1832.56		0.57/2390.00	

#### Comparison against the best known solutions

As it was mentioned, the literature devoted to the MDFSMVRP is rather scarce and the published works on this specific variant are focused on heuristic methods. We are only aware of the exact bounds recently obtained by the 3-h CPLEX execution of Salhi et al. (2014). The performance of the proposed formulations is assessed with respect to the available lower bounds provided in Salhi et al. (2014) and to the best upper bounds given heuristically by Vidal et al. (2014). Table 6 presents the results of the best formulations proposed in Sect. 2 compared to the state-of-the-art methods. For completeness, we have also reported the percentage gap between the best LBs and UBs obtained over the proposed formulations in the column *Best gap (%)*. The results in Table 6 show that the proposed formulations could often identify a feasible solution for all the instances, even for the largest instance considered with nine depots and 360 customers, unlike the exact solution method of Salhi et al. (2014). The largest instance solved by this method includes four depots and 100 customers. Models F3 and F4 yield better optimality gaps than that work on all instances. Note also that the improvement with respect to the bounds given by Salhi et al. (2014) are significant. We have improved all the LBs and UBs with respect to that work. The average LB is increased by 8.63% for the first eleven instances solved by Salhi et al. (2014), and the average UB is reduced by 21.30%. One particular example is that of instance 3-85-160 for which the gap was 47.29% and is now just 2.13%. The average gap over all the instances of the first testbed has decreased from 51.00 to 17.84%. The comparison of our best results against the heuristic of Vidal et al. (2014) show that we could not improve the UBs found heuristically but our gaps are tight. Even though the quality of the UBs is not improved, the introduction of these different formulations helps providing very good lower bounds.

**Table 6:** Comparison of the best formulations with the state-of-the-art methods

Instance	Vidal et al. (2014)	Salhi et al. (2014)			Formulation F3			Formulation F4			Formulation F5			F3, F4, F5
	UB	UB	LB	Gap (%)	UB	LB	Gap (%)	UB	LB	Gap (%)	UB	LB	Gap (%)	Best gap (%)
4-55-100	–	1621.90	1299.80	19.86	1438.50	1328.99	7.61	1408.25	1361.85	3.29	**1398**.**30**	**1384**.**55**	0.98	0.98
3-85-100	–	2677.80	1996.00	25.46	2209.95	2040.45	7.67	2163.63	2086.32	3.57	**2131**.**46**	**2116**.**12**	0.72	0.72
3-85-160	–	2516.00	1326.10	47.29	1675.05	1360.58	18.77	1663.01	1423.30	14.41	**1483**.**51**	**1451**.**86**	2.13	2.13
4-50-80	**1477**.**73**	1725.20	1318.50	23.57	1573.68	1348.51	14.31	1565.27	1390.51	11.16	1733.20	**1416**.**09**	18.30	9.53
4-50-160	**957**.**73**	1378.90	799.70	42.00	1021.59	832.21	18.54	1122.08	**907**.**71**	19.10	1217.74	902.18	25.91	11.14
5-75-140	**1569**.**67**	2561.10	1341.40	47.62	1831.02	1388.41	24.15	1828.73	**1483**.**11**	18.92	NF	NF	–	18.90
2-100-100	**2292**.**64**	3039.70	2079.30	31.59	2660.50	2116.36	20.45	2725.87	2201.16	19.25	2757.95	**2236**.**91**	18.89	15.92
2-100-200	**1453**.**64**	2265.60	1228.30	45.78	1818.69	1280.61	29.59	1825.91	**1396**.**05**	23.54	NF	NF	–	23.23
3-100-100	**2208**.**66**	3390.30	1970.20	41.89	2648.17	2013.95	23.95	2696.05	**2109**.**31**	21.76	NF	NF	–	20.34
4-100-100	**2198**.**91**	3609.90	1971.30	45.39	2626.33	2003.95	23.70	2684.89	**2104**.**36**	21.63	NF	NF	–	19.87
2-80-60	**2072**.**18**	2846.40	1607.60	43.52	2566.13	1683.51	34.39	2565.53	1788.35	30.29	2571.35	**1794**.**38**	30.22	30.05
4-160-60	**3973**.**47**	NF	3032.60	–	5157.49	3094.10	40.01	5172.65	**3506**.**89**	32.20	NF	NF	–	32.00
6-240-60	**5887**.**43**	NF	NF	–	7758.97	4564.18	41.18	7758.97	**5243**.**12**	32.43	NF	NF	–	32.42
9-360-60	**8709**.**26**	NF	NF	–	11638.50	0.00	100.00	11638.50	**7852**.**44**	32.53	NC	NC	–	32.53
Average gap				51.00			28.88			20.63			56.94	17.84

Bold values indicate the new best results

**Table 7:** Average percentage gaps with and without valid inequalities

Instances	Formulation F1		Formulation F2		Formulation F3		Formulation F4		Formulation F5	
	Without VI	With VI	Without VI	With VI	Without VI	With VI	Without VI	With VI	Without VI	With VI
Existing	94.66	90.99	61.79	61.65	29.45	28.88	25.52	20.29	62.43	56.94
New	57.68	21.76	40.76	38.82	2.05	1.64	0.69	0.49	1.18	0.57

**Table 8:** Average percentage gaps for different configurations of formulations F3, F4, and F5

	Average gap	Average gap	Average gap
	F3	F4	F5
Without any VI	29.45	25.52	62.43
Without (40)–(42)	28.93	25.50	–
With all VI	28.88	20.29	56.94
With VI (56)–(58)	–	–	57.43
With VI (59)–(61)	–	–	57.78

#### Effect of valid inequalities

We now briefly analyze the effect of the valid inequalities proposed for each model. We study the effect of valid inequalities in each model on the gap. We have decided not to study the impact of each valid constraint proposed in each model because this would lead to a combinatorial and unmanageable comparison between valid inequalities, which is not the focus of this paper. We have compared the average gaps between two configurations of each formulation, without and with valid inequalities, on each testbed, with the maximum computing time limit of 3 h. Table 7 summarizes these results. On average, they clearly show the benefits of using valid inequalities especially for the explicit formulation. The average gap of model F1 is reduced by almost 50% on the new smaller instances. We can also observe that the introduction of valid inequalities is more relevant for formulations which explicit the index of the vehicle because it is hard to generate efficient valid inequalities for variables that do not carry at least the vehicle type traversing an arc, as it is the case of formulation F3.

We have also reported the effect of some families of valid inequalities, and the average gap yielded by models F3, F4, and F5. Specifically, we have assessed the performance of routing constraints (40)–(42) which have been adapted from Koç et al. (2016a) to formulations F3 and F4. Table 8 shows that these new configurations substantiate the low efficiency of these constraints especially for the commodity flow formulation, with aggregated flow variables. Table 8 shows also the effect of capacity constraints (56)–(58) and of routing inequalities (59)–(61) for model F5. It shows that their introduction improves the performance, reducing the average gap from 62 to 57%. The combination of both sets of inequalities yields an average gap of 56.94%.

## Conclusions

In this paper we have modeled and solved the MDFSMVP. We have presented five different formulations for this difficult distribution problem. The first one is a three-index VRP formulation with an explicit vehicle index, and the second one is more compact, in which individual vehicles are not explicitly identified. The third and the fourth models are commodity flow formulations without a vehicle index. They are based on loading variables to model capacity and connectivity requirements. The fifth and last model is a capacity-indexed formulation, which is a based more compact single commodity flow. We have also provided a survey on how these formulations relate to each other from a theoretical perspective. We have proposed several valid inequalities to strengthen the formulations and we have solved them by branch-and-cut and by branch-and-bound.

We compared the bounds of these formulations on existing instances and on newly generated ones. The results show that the commodity flow formulations and the capacity-indexed formulation provide better bounds. Our results also show that compact formulations represent a very promising research avenue. On classical benchmark instances our methods could improve all previous lower bounds, and we have obtained the best upper bounds and gaps when compared to another exact method from the literature.
